# Geographic variation in breeding system and environment predicts melanin-based plumage ornamentation of male and female Kentish plovers

**DOI:** 10.1007/s00265-015-2024-8

**Published:** 2015-10-24

**Authors:** Araceli Argüelles-Ticó, Clemens Küpper, Robert N. Kelsh, András Kosztolányi, Tamás Székely, René E. van Dijk

**Affiliations:** Department of Biology and Biochemistry, University of Bath, Claverton Down, Bath, BA2 7AY UK; Department of Animal and Plant Sciences, University of Sheffield, Western Bank, Sheffield, S10 2TN UK; Department of Ecology, Faculty of Veterinary Science, Szent Istvan University, Rottenbiller u. 50, H-1077 Budapest, Hungary; MTA-DE “Lendület” Behavioural Ecology Research Group, Department of Evolutionary Zoology, University of Debrecen, Egyetem tér 1, H-4032 Debrecen, Hungary; Institute of Zoology, University of Graz, Universitätsplatz 2, 8010 Graz, Austria

**Keywords:** Ornamentation, Sexual selection, Breeding system, Kentish plover, Melanin

## Abstract

Sexual selection determines the elaboration of morphological and behavioural traits and thus drives the evolution of phenotypes. Sexual selection on males and females can differ between populations, especially when populations exhibit different breeding systems. A substantial body of literature describes how breeding systems shape ornamentation across species, with a strong emphasis on male ornamentation and female preference. However, whether breeding system predicts ornamentation within species and whether similar mechanisms as in males also shape the phenotype of females remains unclear. Here, we investigate how different breeding systems are associated with male and female ornamentation in five geographically distinct populations of Kentish plovers *Charadrius alexandrinus*. We predicted that polygamous populations would exhibit more elaborate ornaments and stronger sexual dimorphism than monogamous populations. By estimating the size and intensity of male (*n* = 162) and female (*n* = 174) melanin-based plumage ornaments, i.e. breast bands and ear coverts, we show that plumage ornamentation is predicted by breeding system in both sexes. A difference in especially male ornamentation between polygamous (darker and smaller ornaments) and monogamous (lighter and larger) populations causes the greatest sexual dimorphism to be associated with polygamy. The non-social environment, however, may also influence the degree of ornamentation, for instance through availability of food. We found that, in addition to breeding system, a key environmental parameter, rainfall, predicted a seasonal change of ornamentation in a sex-specific manner. Our results emphasise that to understand the phenotype of animals, it is important to consider both natural and sexual selection acting on both males and females.

## Introduction

‘Hence that male which at [the selection] time is in fullest vigour, or best armed with arms or ornaments of its species, will gain in hundreds of generations some small advantage and transmit such characters to its offspring…’ (Darwin 1842). Darwin (1842) already realised that selection by females on male ornaments may drive male morphological evolution. He also acknowledged that the same principle may apply to females (Darwin 1871). However, understanding the function of a phenotype and how a certain phenotype may evolve remains a fundamental aim of sexual selection research. Prime candidate phenotypes in which to investigate these questions are ornaments used in sexual displays. Consistent individual differences in the elaboration of such ornaments may signal attractiveness of an individual, and more attractive individuals are often more successful in obtaining additional partners and thus less likely to provide parental care (Magrath and Komdeur 2003; Houston et al. 2005; van Dijk et al. 2012). Hence, mating success and parental care are intrinsically connected as important aspects of breeding systems (Owens and Bennett 1997; Thomas and Székely 2005; Alonzo 2010) and are often associated with ornamentation. Consequently, breeding systems may be expected to drive the evolution of ornamentation.

Variation in breeding opportunities is likely to determine to what extent ornamentation should predict mating success and parental care. With increasing breeding opportunities, sexual selection on traits that enhance mating success, i.e. traits signalling attractiveness, should intensify (Wiklund and Forsberg 1991; Gonzalez-Voyer et al. 2008; Bedhomme et al. 2009). This in turn may be determined by the environment influencing sex differences in, for example, the costs of producing ornaments or of being deserted (Kvarnemo and Ahnesjo 1996) and the benefits of choosiness (e.g. ‘good parent hypothesis’; Heywood 1989; Hoelzer 1989; Owens and Thompson 1994). Breeding opportunities are an important determinant of this balance of costs and benefits of choosiness. As a result, selection should act more strongly on the sex with more breeding opportunities (males in most species, which can often secure more than one female; Arnold and Duvall 1994). Mating systems are thus often associated with the extent of male and female ornamentation, so that plumage dimorphism is largest in polygynous or lek species, compared to species with any other mating system, including polyandrous species. Therefore, the fact that males exhibit more elaborate ornaments in polygynous and lek species as a result of increased intensity of sexual selection, while males and females exhibit drabber plumage in species with other mating systems, appears to be an important explanation behind differences in sexual dimorphism observed between species (Dunn et al. 2001).

In addition to social selection pressures influencing the divergence of phenotypic variation, different ecological settings also play an important role in the evolution of sexual traits (Andersson 1994; Mobley and Jones 2009; McGraw et al. 2010). Breeding system variation displayed both within and between populations is likely to result from the coevolution of reproductive behaviours of males and females within an ecological setting (Emlen and Oring 1977; Davies 1991), which may directly explain the variance in sexually selected traits across a species’ range (Cockburn et al. 2008; Roulin et al. 2009, 2011). For example, barn owls (*Tyto alba*) living in the tropics display larger eumelanic spots than those found in temperate zones, which is possibly due to a higher abundance of parasites in the tropics, since individuals with larger melanin-based spots are more resistant to ectoparasites (Roulin 2004; Roulin et al. 2009). The environment may also indirectly influence ornament development and expression through the breeding system. Examining regional, or seasonal, differences in the expression of sexually selected traits is thus important to understand sexual selection (Galván and Moreno 2009), because local environmental conditions are likely to influence the trade-off between natural (costs of ornamentation) and sexual (benefits) selection (Hegyi et al. 2002, 2006).

Although secondary sexual characters are widespread in females, research on sexual selection has focused almost exclusively on selection on elaborate traits in males (Clutton-Brock 2007). However, in order to obtain a full understanding of how ornaments may evolve, geographic variation in sexual dimorphism needs to be investigated (Amundsen 2000; Chui and Doucet 2009). In recent years, a growing interest in trait elaboration in females has emerged (Clutton-Brock 2007, 2009; Edward and Chapman 2011). Yet despite this, we do not fully understand the role of female ornaments or the evolutionary forces maintaining them (Hegyi et al. 2008). It is unclear whether the same underlying principles and mechanisms that commonly operate in males also apply to females (Amundsen 2000; Rubenstein and Lovette 2009).

In birds, melanin-based ornamentation often plays an important role in both male-male competition and in inter-sexual interactions (Kingma et al. 2008; Chaine et al*.*^2013^; Da Silva et al*.*^2013^). An important characteristic of sexually selected ornaments is that they should be costly to the bearer (Olson et al. 2008; Jennions and Kokko 2010). The costs of melanin ornaments may consist of the physiological costs of the ornaments, which are often related to circulating levels of androgens (Bókony et al. 2008), costs in terms of time and energy (including the risk of injuries) related to competitive interactions over social status and costs associated with conspicuousness towards potential predators (Jawor and Breitwisch 2003; Ekanayake et al. 2015). However, the adaptive value of melanin-based ornamentation in terms of mate choice remains poorly understood. Although a number of studies have indicated that melanin-based ornaments may be reliable signals of good genes or of parental quality (Niecke et al. 2003; Bókony and Liker 2005; Dunn et al. 2008), others have shown covariation of melanin-based colouration with costly traits (e.g. Fernandez and Morris 2008). Such costs may be maintained by frequency- or condition-dependent selection, local adaptation or pleiotropy. The expression of pleiotropic genes, which may simultaneously regulate melanogenesis and, for example, body condition, may vary depending on the environment (Ducrest et al. 2008; Fernandez and Morris 2008; Dall et al. 2015; Roulin 2015).

Here, we investigate whether the elaboration of melanin-based ornamentation is predicted by breeding systems and environment, using data on ornament variability, breeding system and environmental conditions from five geographically distinct populations of Kentish plover, *Charadrius alexandrinus*. This precocial shorebird is particularly suitable for this purpose, because of its uniquely diverse breeding system. Firstly, Kentish plovers exhibit diverse mating systems and variable parental care both within and across populations: after the eggs hatch, either the male or the female, or neither, may desert the brood and find a new mate (Székely and Lessells 1993; Fraga and Amat 1996; Kosztolányi et al. 2006; Vincze et al. 2013). Secondly, this species is sexually dimorphic. Males tend to have distinctive black ear coverts, a horizontal head bar, an incomplete breast band (i.e. one black band on each side of their breast) and a rufous crown, while the females tend to be more drab (Székely et al. 1999). Male Kentish plovers display their breast feathers to females or to other males during courtship and agonistic encounters (Perrins 1998; Kis and Székely 2003). Lendvai et al. (2004a, b) showed that large-badged males may have an advantage in aggressive male-male encounters and that the breast band size of males is related to the volume of their clutches. Similar findings have been published for various passerine birds, showing that the size of the breast band is a reliable indicator of genetic quality (e.g. Møller 1988; Norris 1993; Bouwman et al. 2007). This suggests that breast bands signal mate attractiveness or dominance and are thus a candidate trait for sexual selection to act upon.

Despite great interest in sexual selection, relatively little is known about the different determinants of secondary sexual characters in natural populations. The aim of this study was to gather such information to determine how ornamentation and sexual dimorphism within geographically distinct populations of a species may respond to sexual and natural selection. We investigate the extent to which sexual plumage dimorphism differs across five populations. Because the variance of reproductive success generally increases with increasing levels of polygamy (Björklund 1990; Bedhomme et al. 2009), we predicted that in populations with higher levels of polygamy, where sexual selection on males is more intense, males should exhibit more elaborate ornamentation compared to monogamous populations. Secondly, because sexual selection on males is predicted to be higher in polygamous than in monogamous populations, we predicted that sexual dimorphism should be more pronounced in polygamous populations.

Thirdly, we predict that the association of male and female ornamentation with breeding system will be moderated by the environment. Various pathways leading to the expression of ornaments are expected to be influenced by the environment. In addition, colouration may be affected by pleiotropic effects, resulting in different phenotypes due to a change in the underlying genetic components (Gratten et al. 2008). This means that selection on melanin-based colouration may be conditional to the environment (Dall et al. 2015; Roulin 2015). Furthermore, because sexually selected ornaments are expected to be costly to the bearer (Olson et al. 2008; Jennions and Kokko 2010), adverse environmental conditions should suppress the expression of costly ornaments, because individuals will be less able to produce, maintain or exhibit elaborate ornaments. Kentish plovers often feed on the shoreline in invertebrate-rich moist-soil areas which show lower prey abundance and diversity in longer dry periods (Anderson and Smith 2000). If food is sparsely available in populations with low levels of rainfall, ornaments are predicted to be smaller and lighter than in populations with high rainfall. However, the potentially complex underlying quantitative genetics of ornamentation make it difficult to pose clear predictions as to how ornamentation is related to the environment and whether, for instance, food availability or genetics mediate ornamentation through a change in environment. Here, we aim to explore whether environment, i.e. rainfall during the breeding season, is associated with melanisation independent of or in concert with breeding system.

## Materials and methods

### Study sites and general methods

**Fig. 1:**
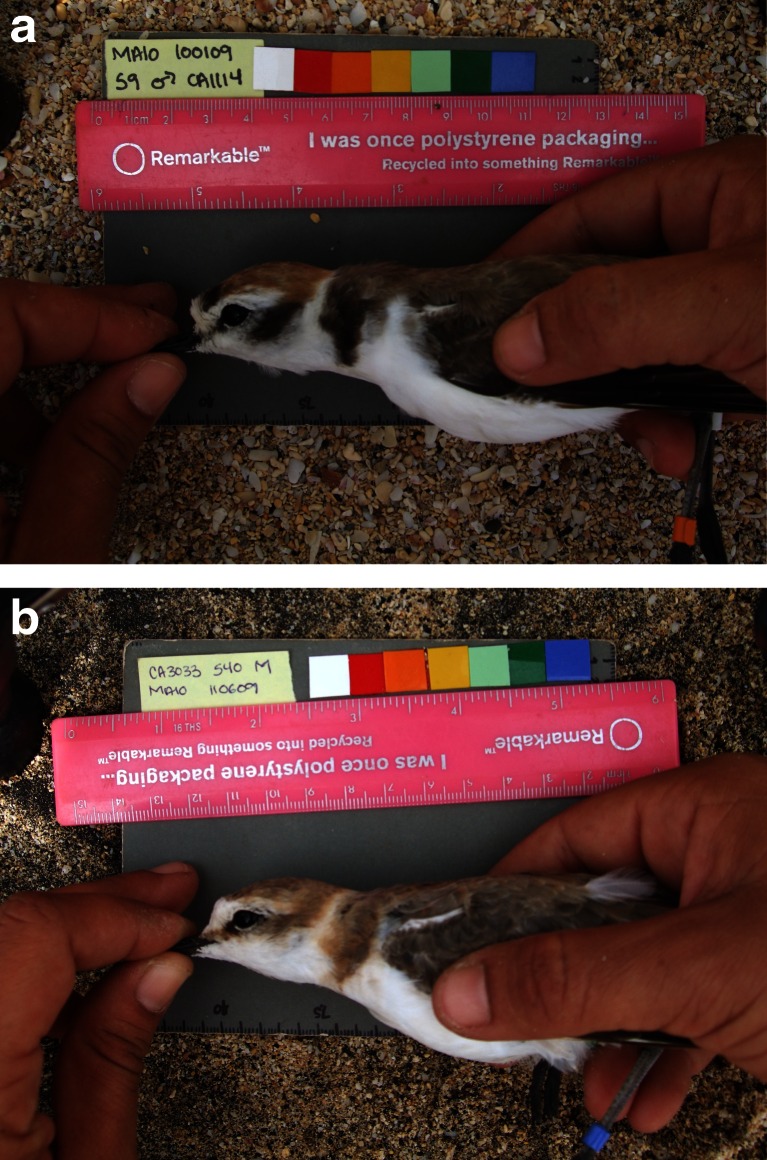
Examples of photographs used for taking measurements of ornament size and brightness. Displayed here are examples of male Kentish plovers from Maio, Cape Verde, with **a** large, dark ornaments and **b** small, pale ornaments

We studied breeding adults in five geographically distinct populations of Kentish plover for which detailed information on breeding systems is available (Table 1). Adult plovers were captured using funnel traps during incubation and after the first 5 days of incubation. This was done within 42.2 ± 20.9 days (mean ± SD, range 10–64 days) at all populations. All birds were ringed with one numbered metal ring and an individual combination of colour rings, and tarsus length was measured (to the nearest 0.1 mm). Additionally, we took at least one digital photograph of the left and the right side of the plovers (Table 1). These photographs were taken by different observers using standardised methods (Fig. 1). In short, the camera was mounted on a tripod or held by a second observer at a height of approximately 50 cm. Individuals were photographed, against a neutral grey background (Kodak 18 %) including a scale reference, using a Nikon Coolpix 4500 (Tuzla, Doñana and Al Wathba) or a Fuji Finepix F40 (Maio and Farasan Island). The bird was positioned touching the grey card while it was held with two hands so that the neck was stretched in a horizontal and straight-line position.

**Table 1:** Sampling location and breeding system for populations of Kentish plover, the year when the photographs were taken (median and range of capture dates), the number of photographs (*n*_p_), the number of individuals (*n*_i_; number of males and females, respectively, in parentheses) from each site included in the analyses and the average monthly rainfall (mm)

Population	Breeding system description	Year	*n*_p_	*n*_i_	Rainfall
Polygamy					
Tuzla (Turkey)	Sequential polygamy with higher remating opportunity for females than males (Székely et al. 1999). Uniparental care by males is more common than uniparental care by females (Kosztolányi et al. 2006)	2010 (4 Jun; 12 May–22 Jun)	164	41 (20, 21)	24.0
Doñana National Park (Spain)	Sequential polygamy (average 32 % of individuals polygamous, range 0–64 %). Higher remating opportunity for females than males. Uniparental care by male and biparental care (Amat et al. 1999)	2004 (1 Jun; 14 Apr–17 Jun)	188	94 (45, 49)	22.2
Al Wathba (United Arab Emirates)	Sequential polygamy. Uniparental and biparental care by male or female co-occur (Kosztolányi et al. 2009).	2005 (20 May; 1 May–9 Jun)	165	32 (15, 17)	0.0
Monogamy					
Farasan Island (Saudi Arabia)	Monogamy. Biparental care (Alrashidi et al. 2011)	2009 (29 Jun; 23 Jun–3 Jul)	104	26 (12, 14)	3.4
Maio (Cape Verde)	Monogamy. Biparental care and resident life-history strategies (Argüelles-Ticó 2011)	2008 and 2009 (16 Oct. 20 Sep–16 Nov)	858	143 (70, 73)	30.1

We obtained monthly rainfall data for a 5-km radius for each population from Worldclim (http://www.worldclim.org) at a 0.5′ resolution. These rainfall data were extracted from Worldclim for each year and month in which the population was studied, and monthly rainfall data were assigned to the month in which individuals were captured in each population, so that a given population may have multiple estimates of rainfall if individuals were captured over more than 1 month. Spatial analyses were performed in ArcGIS. The amount of rainfall was used as our proxy for availability of food and humidity in our analyses.

### Ornament measurements

We measured colour and size of four melanin-based plumage patches on the frontal body region of the Kentish plovers (Bókony et al. 2003; Lendvai et al. 2004a): the dark, lateral patches on either side of the breast (‘breast bands’) and the ear coverts. All images were imported into Adobe Photoshop (version 7.0 for Windows) and calibrated for white balance and size using the grey card and ruler, respectively. The size (in cm^2^) of the breast bands and the ear coverts was determined by counting the number of pixels in each patch, using the ‘rectangular marquee’ and the ‘magic wand’ tool, the latter set for a fixed tolerance level (20 %), and the selected area was manually adjusted to contain the melanin patch of interest only. Setting the limits of areas was facilitated by the abrupt and regular transition between these plumage patches and the differently coloured adjacent plumage tracts, although this transition is less pronounced for lighter coloured ornaments (Fig. 1). We then measured the median red, green, blue (RGB) values for each patch selected, using the histogram command. These RGB values were then used to calculate the brightness of all patches (a value between 0 and 100; Pascale 2010). Brightness is used here to refer to the achromatic notion of intensity (Pascale 2010). Size and brightness scores of breast bands and ear coverts were calculated by averaging the values recorded for the left and right sides of all pictures available for each individual taken on the same day at a single capture event. The breeding plumage of Kentish plovers does not show UV reflection (AAT, pers. obs.), while no support for UV sensitivity in Charadriidae has been found (Ödeen et al. 2010). All photographs were analysed by one observer (AAT), except for Cape Verde, where there was a second observer (R. Smart). Due to the nature of the photographs (Fig. 1) and organisation of the photographs and data, these observers were not blind to the population of origin of the birds. These observers were blind to the sex of the birds at the time of analysis but not to the population of origin. Ornament size and brightness were highly repeatable within observers (*n* = 20 individuals, *r* > 0.823, *P* < 0.001) and between observers (*n* = 12 individuals, *r* > 0.852, *P* < 0.001; Harper 1994). Tarsus length, a proxy variable for body size, was not associated with any of our ornament measurements (*P* > 0.180), but was included as a covariate in all models to correct for body size (Green 2001).

### Statistical analyses

Populations were grouped in two categories of mating system, monogamy or polygamy, based on published information from papers or theses (Table 1). For Farasan Islands and Al Wathba, no information on mating system was available and we therefore used parental care as a proxy for mating system since there is a tight correlation between mating and parental care system in shorebirds (Székely et al. 2007). For Doñana, no data on parental care or mating system are published although a detailed study was conducted on a nearby Kentish plover population (Fuente de Piedra, <150 km away; Amat et al. 1999) which is well within the range of breeding dispersal of polygamous plovers (median 145–180 km; Stenzel et al. 1994) and therefore mating system data were adopted from this population for the nearby Doñana population.

Variables describing ornamentation were multi-colinear. We therefore carried out a principal component analysis (PCA) and reduced the number of variables describing ornamentation by extracting two principal components (PCs) using varimax rotation with Kaiser normalisation (Table 2). These PCs were subsequently used in all analyses. The two principal components (PC1 and PC2) explained 76.6 % of the total variation in ornamentation (47.8 and 28.8 %, respectively). PC1 (henceforth ‘brightness’) primarily accounted for ornament brightness, such that low scores corresponded with darker plumage and higher scores with lighter plumage. PC2 (henceforth ‘size’) described the size of the patches, with larger values corresponding to larger ornaments (Table 2).

**Table 2:** Factor loadings of each variable of ornamentation and explained variances from a principal component analysis of brightness and size of the breast bands and ear coverts in different Kentish plover populations (*n* = 336 individuals)

	Brightness (PC1)	Size (PC2)
Breast band brightness	0.930*	−0.053
Ear covert brightness	0.923*	−0.139
Breast band size	0.002	0.832*
Ear covert size	−0.176	0.776*
Eigenvalue	1.911	1.152
% variance accounted for	47.78	28.81

Values of factor loadings >0.7 are in italics

*P < 0.001

We used linear mixed models (LMMs) in the package nlme for R (Pinheiro et al. 2010) to account for the statistical non-independence of data originating from a given population. Our LMMs to analyse ornamentation in response to breeding system and monthly rainfall included the PCs for brightness and size of ornaments as response variables, an interaction between sex and breeding system, an interaction between the date the picture was taken (henceforth ‘capture date’) and the amount of rainfall and an interaction between sex and rainfall as predictor variables, while sex and breeding system were included as fixed factors, rainfall and capture date as covariates and population as the random factor. The interaction between capture date and rainfall was included because a change in ornamentation over the season may depend on the amount of precipitation, which may influence wear, the importance of protection against feather-degrading bacteria (Gunderson et al. 2008) and food availability. The interaction between sex and rainfall was included to assess whether environmental conditions may limit ornament expression in one sex, but not in the other, which would be expected if there is directional selection on ornamentation in one of the sexes. However, we found that this interaction did not contribute significantly to either the LMM for analysis of ornament brightness (model effect estimate ± SE = 0.010 ± 0.007, *t* = 1.475, *P* = 0.141) or the LMM for ornament size (0.006 ± 0.006, *t* = 1.064, *P* = 0.288), and was thus removed from our final model. Capture date was calculated as the number of days since 1 March, after which it was standardised for each population by subtracting the mean capture date from each capture date value and dividing that by the standard deviation for each population (i.e. the standard deviate was calculated, Sokal and Rohlf 1995). In order to assess the effect of lost data variance in our analysis due to conducting a PCA, we also performed an additional analysis on untransformed, original data on breast band brightness only. This model included the same variables as the mixed model above, using breast band brightness as the response variable. We chose breast band brightness, because the residuals from the mixed models on ornament size were not normally distributed, while the breast bands are the largest ornamental plumage patch of Kentish plovers (Fig. 1) and brightness, not size, likely the best ornamental indicator of the intensity of sexual selection in this species (see ‘Discussion’). All random effects were fitted as random intercepts. For plovers that were captured several times within the same season or between seasons (*n* = 4), only one randomly selected datum per individual was included in all analyses to avoid pseudoreplication. Sample sizes vary between analyses due to missing values. PCAs were performed using SPSS version 16.0 for Windows, and all other statistical analyses were done using R version 2.11.1.

## Results

### Ornaments and breeding system

**Fig. 2:**
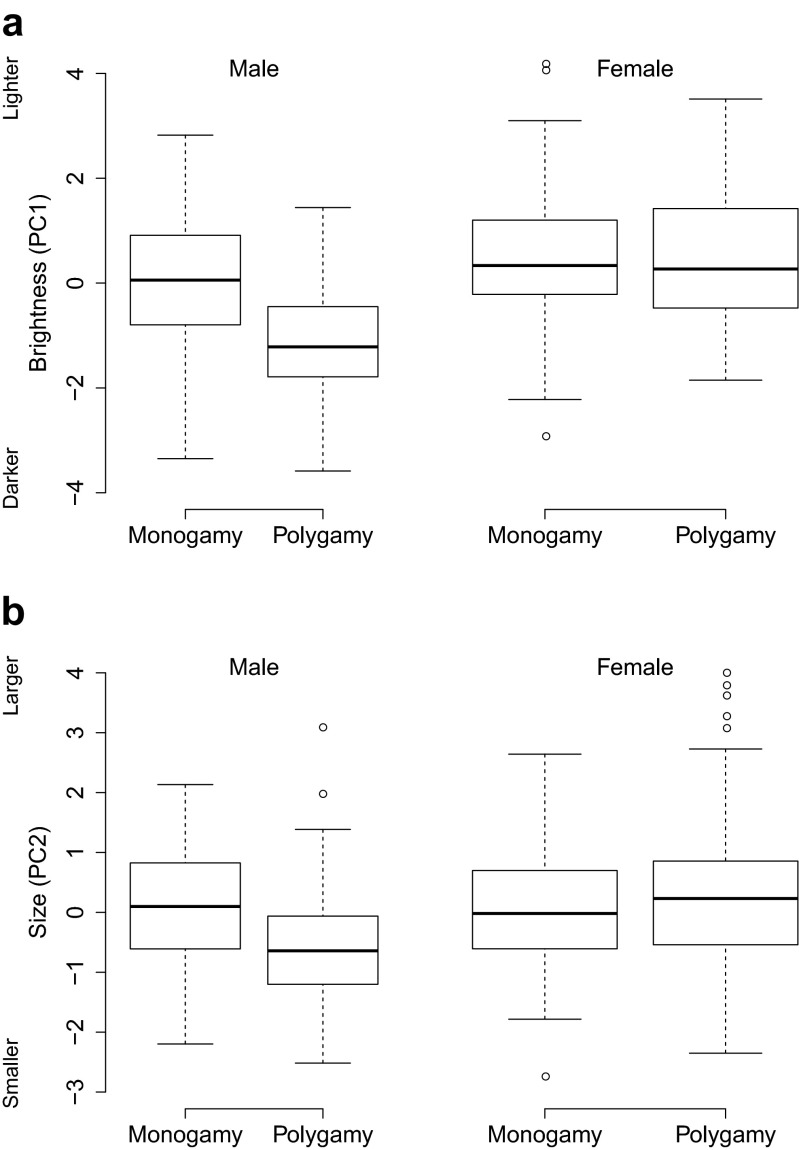
Box plots of **a** brightness and **b** size of male and female ornaments in relation to breeding system. Box plots indicate the median, the inter-quartile range, the maximum and minimum values excluding outliers and outliers. Outliers are defined as data points that are outside 1.5 times the inter-quartile range from the first and third inter-quartiles

**Fig. 3:**
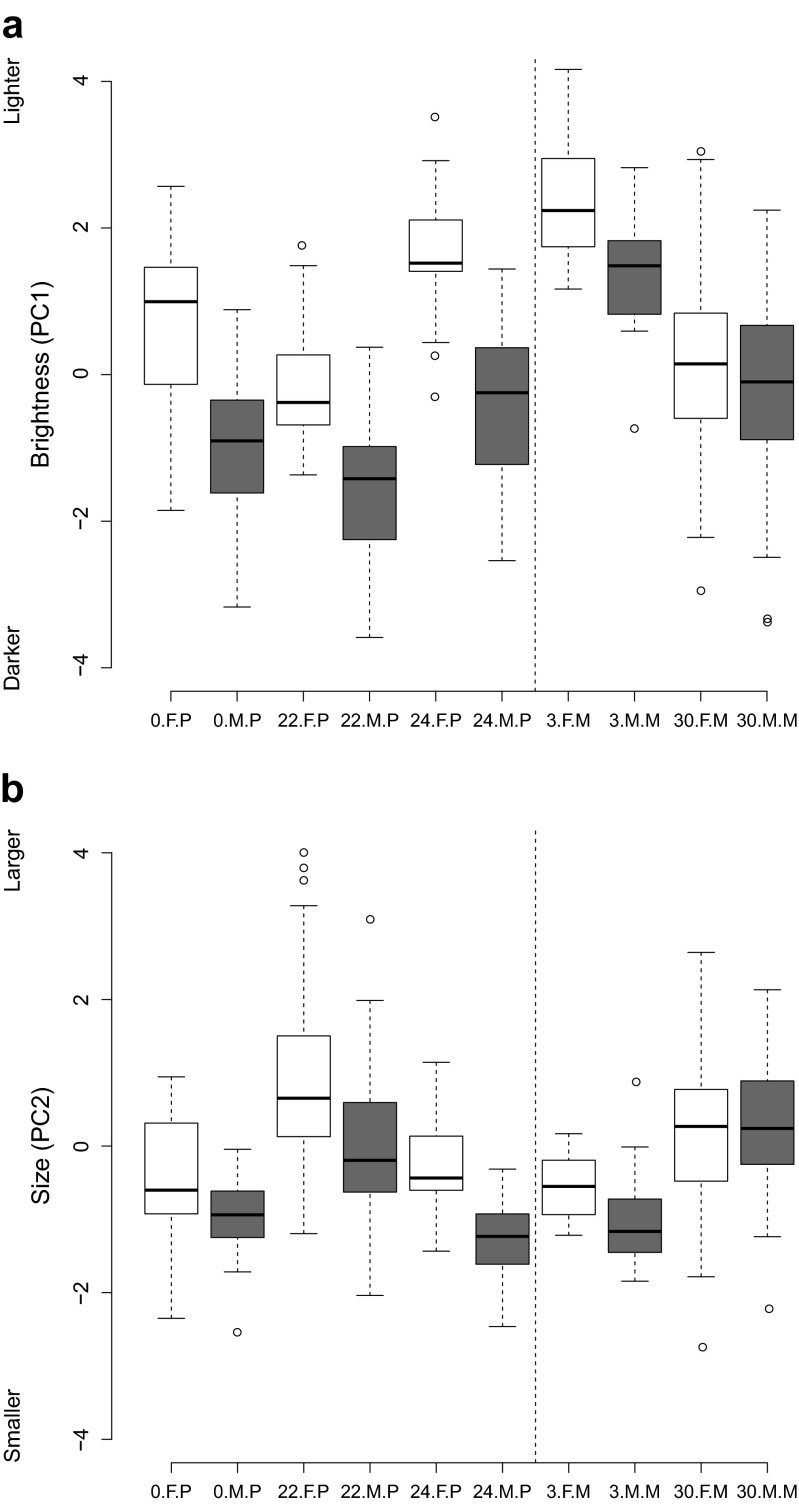
Box plots of **a** brightness and **b** size of male and female ornaments in relation to breeding system and rainfall for each population separately. Values on the *x-axis* indicate the following: the *first character* indicates the mean rainfall (mm) during the study period per population, the *second character* is the sex (*F* = female, *M* = male) and the *third character* indicates the breeding system (*P* = polygamous, *M* = monogamous). Values for females are depicted as *white boxes*, those for males as *grey boxes. Box plots left of the dashed line* are for polygamous populations, and *box plots right of the dashed line* for monogamous populations. Box plots indicate the median, the inter-quartile range, the maximum and minimum values excluding outliers and outliers. Outliers are defined as data points that are outside 1.5 times the inter-quartile range from the first and third inter-quartiles

We analysed 1479 photographs of 336 individuals (174 females and 162 males) from five geographically distinct populations (Table 1). Breeding system was associated with ornamentation of males and females in different ways, indicated by significant interactions between breeding system and sex for both brightness and size of the ornaments (Table 3, Figs. 2 and 3). Males exhibited darker and smaller ornaments in polygamous compared to monogamous populations (Figs. 2 and 3; Table 4). Females, however, exhibited larger ornaments in polygamous than in monogamous populations, while there was no clear difference in brightness of female ornaments between polygamous and monogamous populations (Fig. 2 and 3; Table 4). Ornamentation of males and females within pairs was correlated across populations (brightness: *S* = 127,900, *P* < 0.001, *ρ* = 0.468, *n* = 133; size: *S* = 177,000, *P* = 0.005, *ρ* = 0.264, *n* = 133).

**Table 3:** Linear mixed models (LMM) of brightness and size of ornaments as predicted by sex, breeding system and rainfall (*n* = 304). Model effect estimates ± SE are given. Factor levels included in the intercept of both models are ‘female’ for the factor ‘sex’ and ‘monogamous’ for the factor ‘breeding system’

	Brightness				Size			
	MEE ± SE	*df*	*t*	*P*	MEE ± SE	*df*	*t*	*P*
Intercept	3.424 ± 1.714	293	1.998	0.047	−1.179 ± 1.433	293	−0.822	0.412
Sex	−0.410 ± 0.169	293	−2.430	0.016	−0.064 ± 0.142	293	−0.447	0.655
Breeding system	−0.428 ± 0.904	3	−0.474	0.668	0.349 ± 0.730	3	0.478	0.666
Rainfall	−0.001 ± 0.007	293	−0.076	0.939	−0.017 ± 0.006	293	−2.797	0.006
Capture date	0.280 ± 0.099	293	2.842	0.005	0.192 ± 0.083	293	2.316	0.021
Tarsus length	−0.081 ± 0.055	293	−1.478	0.141	0.038 ± 0.046	293	0.834	0.405
Sex*breeding system	−1.163 ± 0.239	293	−4.870	<0.001	−0.886 ± 0.201	293	−4.406	<0.001
Rainfall*capture date	−0.006 ± 0.003	293	−1.819	0.070	−0.009 ± 0.003	293	−3.150	0.002

**Table 4:** Descriptive statistics of the brightness and size of male and female ornaments per population. Means ± SDs (range) are provided of the mean ornament brightness and of the total ornament size of ear coverts and breast bands of Kentish plovers

	Brightness	Size
Males		
Tuzla (Turkey)	43.8 ± 7.9 (27.3–58.2)	1.29 ± 0.23 (0.90–1.71)
Doñana National Park (Spain)	33.6 ± 6.9 (18.2–48.4)	1.89 ± 0.53 (0.89–3.94)
Al Wathba (United Arab Emirates)	39.2 ± 8.2 (20.6–54.3)	1.45 ± 0.31 (0.83–1.95)
Farasan Island (Saudi Arabia)	57.1 ± 7.6 (40.4–69.0)	1.43 ± 0.39 (0.95–2.38)
Maio (Cape Verde)	43.9 ± 9.3 (19.3–62.8)	1.96 ± 0.44 (1.07–3.06)
Females		
Tuzla (Turkey)	59.0 ± 7.1 (43.9–72.7)	1.70 ± 0.36 (1.04–2.32)
Doñana National Park (Spain)	42.8 ± 5.8 (33.5–60.2)	2.43 ± 0.71 (1.21–4.41)
Al Wathba (United Arab Emirates)	52.4 ± 9.3 (32.4–67.1)	1.68 ± 0.42 (0.98–2.54)
Farasan Island (Saudi Arabia)	64.6 ± 7.2 (54.2–78.0)	1.59 ± 0.27 (1.04–1.99)
Maio (Cape Verde)	46.9 ± 8.8 (21.9–68.8)	1.92 ± 0.50 (0.54–3.31)

### Ornaments and environment

We found that the interaction term of rainfall with capture date was significantly associated with the size, and tended to predict brightness of ornaments (Table 3). When we analysed these results for males and females separately, we found that male ornaments appeared to get darker over the course of the season in areas with high rainfall, but in particular got lighter with the advance of the season in areas with low rainfall (−0.012 ± 0.005, *df* = 138, *t* = −2.333, *P* = 0.021, *n* = 147). Brightness of female ornaments was not predicted by the interaction between time of the season and the amount of rainfall (−0.001 ± 0.005, *df* = 148, *t* = −0.312, *P* = 0.755, *n* = 157).

The size of male ornaments tended to increase over the course of the season in areas with high rainfall and to decrease when rainfall was sparse (−0.007 ± 0.004, *df* = 138, *t* = −1.741, *P* = 0.084, *n* = 147). The size of female ornaments depended on the amount of rainfall and the advance of the breeding season in a similar manner and also increased in size over the course of the season in areas with high rainfall and decreased in size in drier environments as the season advanced (−0.012 ± 0.004, *df* = 148, *t* = −2.856, *P* = 0.005, *n* = 157). Crucially, considering the effect of both rainfall and breeding system on male and female ornamentation in one model showed that both the interaction between breeding system and sex and the interaction between the advancement of the breeding season and rainfall are significant predictors of ornamentation (Table 3). We found no evidence that rainfall was correlated with the breeding system: average rainfall per month ranged from 3.4 to 30.1 mm (16.8 ± 18.9 mm (mean ± SD)) in the monogamous populations and from 0.0 to 24.0 mm (15.4 ± 13.4 mm; Table 1) in the polygamous populations (Fig. 3).

We found qualitatively similar results when rainfall was included as a binary factor in these models (i.e. ‘wet’ versus ‘dry’), with the interaction effects of sex*breeding system (*P* < 0.001) and rainfall*capture date both remaining significant predictors in the model. Our results also remained qualitatively similar when we analysed breast band brightness only, i.e. using measured data instead of residuals from a PCA, except that there was no trend for breast band brightness to be predicted by an interaction between rainfall and capture date (−0.030 ± 0.029, *df* = 293, *t* = −1.017, *P* = 0.310). Sex (−5.785 ± 1.451, *df* = 293, *t* = −3.987, *P* < 0.001), capture date (2.470 ± 0.847, *df* = 293, *t* = 2.917, *P* = 0.004) and the interaction between sex and breeding system (−6.923 ± 2.053, *t* = −3.372, *P* < 0.001) were significant predictors of breast band brightness, while breeding system, rainfall and tarsus length were all non-significant variables in the model (*P* > 0.218, *n* = 304).

## Discussion

Variation in the extent of sexual dimorphism is traditionally attributed to differences in mating systems. However, this has mainly been investigated across species (Jones and Avise 2001; Dunn et al. 2001; van Dijk et al. 2010b), while studies addressing the question of how a mating system may shape ornamentation within a species are exceedingly rare. The results from our study investigating geographically distinct populations within one species provide support for patterns similar to those found across species. The differences in both male and female ornamentation in Kentish plovers were associated with variation in mating system, so that populations with a more polygamous breeding system exhibited stronger sexual dimorphism than monogamous populations. These results are consistent with the proposition that the intensity of sexual selection differs across geographically distinct populations with diverse breeding systems. As a result, although data from additional populations are required to corroborate our tentative conclusion, the breeding system appears to have important ramifications for the evolution of male and female ornamentation and sexual dimorphism in Kentish plovers.

We found that the ornaments of males were smaller, but darker, in polygamous compared to monogamous populations. This may be a consequence of the size of ornaments being traded off against their intensity (Hill 1993; Grether 1995). Additionally, the measured size of the ornaments is likely to be influenced by how clearly the ornament can be defined, i.e. lighter, less well-defined patches will be measured as larger, whereas darker, well-defined ornaments may be measured as smaller (Fig. 1). Our results thus suggest that brightness, not size, is the best ornamental indicator of the intensity of sexual selection in this species, although the multiple components of a trait may be evaluated independently and reflect different behavioural or physiological properties of an individual. Moreover, we cannot exclude the possibility that sexual selection would favour smaller melanin-based plumage patches. However, our result that polygamous males exhibited darker ornaments than monogamous ones is consistent with the proposition that, for males, the intensity of sexual selection should be greater in polygamous than in monogamous populations.

We also found that females exhibited smaller ornaments in monogamous compared to polygamous populations, whereas there was no clear difference in brightness of female ornamentation in relation to breeding system. Although sexual selection is expected to act on male traits under polygamy, mechanisms responsible for male ornamentation, such as mutual mate choice, might also influence female ornamentation (Clutton-Brock 2009). Polygamous populations were characterised by a high proportion of female desertion and harboured a significant number of sequentially polyandrous females (Amat et al. 1999; Székely et al. 1999; Kosztolányi et al. 2009). Female ornamentation might thus be under sexual selection and, for example, be used in female-female competition to mate with multiple males. Alternatively, increased female ornamentation may be a result of selection on male ornamentation following genetic correlation between male and female ornamentation (Roulin et al. 2001; Potti and Canal 2011; Kraaijeveld 2014), as reinforced through mutual mate choice (Lande 1980; Amundsen 2000; Kraaijeveld et al. 2007).

We used the social mating system to predict ornamentation. However, the genetic mating system may differ from the social mating system (Birkhead and Møller 1998). Investigations of the genetic mating system of Kentish plovers have been published for only one population (Tuzla), in which extra-pair fertilisations were uncommon (3 % of chicks in total, *n* = 7/229 chicks; Küpper et al. 2004). Similar frequencies have been obtained from other populations (K. Maher et al., unpublished data). Therefore, this species appears predominantly genetically monogamous. It is thus unlikely that the dimorphism observed in populations of Kentish plovers is significantly confounded by extra-pair matings. Additionally, we acknowledge that our analyses are restricted to breeding individuals only and as such may be confounded by a bias in population sampling procedures, because a higher proportion of individuals is expected to breed in monogamous populations compared to polygamous populations. We note, however, that our results concerning breeding males, at least, are consistent with predictions from sexual selection theory.

Male ornaments may play an important role as a badge of status in territorial, aggressive encounters (Rohwer 1975; McGraw et al. 2003; Lendvai et al. 2004a), influencing the trade-off between the costs and benefits of ornamentation. For males, darker (and larger) ornaments should thus be sexually selected. Social status signalling is expected to be relatively important compared to female choice-related signalling, because competition over non-sexual resources is more balanced between the sexes than sexual competition (Kraaijeveld et al. 2007). Additionally, population density is often positively associated with levels of polygamy, because the number of potential future mates, and thus polygamy, available is expected to increase with population density (Kokko and Rankin 2006; McGraw et al. 2010; van Dijk et al. 2010a). However, competition for food and nesting resources may also vary between populations and such variation could mask the effects of sexual selection (Kokko et al. 2004; Alonzo and Sheldon 2010).

### Ornamentation and environment

In addition to breeding system, the evolution of sexually dimorphic signals is known to be influenced by levels of predation, foraging strategies and the background sensory environment, all of which affect the balance between natural and sexual selection (Endler 1978, 1988; Andersson 1994). The fitness conferred by a given signal and the phenotype it symbolises can vary by environment (Qvarnström 2001; Hale 2008; Missoweit et al. 2008), and the environment can influence the expression of colouration genes. This variation could cause sexually selected signals to diverge across populations due to varying local natural selection regimes (Dunn et al. 2008). Our results for breeding system alone may thus be partly confounded by the environment having an important influence on the cost/benefit ratio of ornamentation. We found no evidence for environmental conditions limiting ornament expression more strongly in one sex than in the other. However, we found that the ornamentation changes over the course of the season to become brighter and larger, to an extent correlated with the amount of rainfall. Such change in ornamentation is likely due to wear rather than moult. Nonetheless, there may still be costs associated with such change in ornamentation, for example those generated by aggressive encounters with conspecifics (Jawor and Breitwisch 2003). Soil moistness is associated with food availability (Anderson and Smith 2000), so that with larger amounts of precipitation, food is expected to be more abundant and as a result the costs of ornamentation may be more easily met by the bearer of those ornaments in more humid environments (Evans 1991). This may drive a species to exhibit ornaments for prolonged periods by producing higher levels of melanisation. Accordingly, we found that male ornaments got darker over the course of the season in populations with higher rainfall and lighter in populations with low rainfall.

Additionally, female ornaments increased in size as the season advanced in populations with high rainfall, whereas in populations where rainfall was sparse the size of female ornaments decreased. These results are consistent with Gloger’s rule’, which states that feathers tend to be darker in humid environments than in drier areas (Gloger 1833; Zink and Remsen 1986; Burtt and Ichida 2004; Chui and Doucet 2009). One possible explanation for these patterns is that it may be more important in humid areas to maintain melanin-based ornamentation as it may protect against feather-degrading bacteria (Gunderson et al. 2008). Melanin-based plumage has been observed to change over time due to microbial or ectoparasite activity degrading feather quality (Gunderson et al. 2008). Gloger’s rule may thus mediate the association of ornamentation with breeding system. Moreover, accumulation of dirt, feather abrasion or exposure to ultraviolet light may all be influenced by weather conditions during the breeding season (Delhey et al. 2010) and the extent to which these processes influence ornamentation is thus likely to vary between environments.

The apparent change in ornamentation with the advance of the season we found here may be confounded by differently ornamented individuals breeding at different times of the season (Dreiss and Roulin 2010), which in turn might be influenced by the amount of precipitation. Data on a change in ornamentation from individuals recaptured throughout the breeding season are needed to test this alternative explanation. Yet, our results suggest that the association of male and female ornamentation with climatic conditions may moderate the impact of sexual selection as associated with breeding system. Importantly, however, the association between rainfall and male and female ornamentation was significant after accounting for the effect of breeding system. Indeed, our results suggest that both rainfall and breeding system influence melanin-based plumage ornamentation in Kentish plovers.

In addition to the direct impact the environment may have on ornamentation, the environment may also affect the breeding system, and as such indirectly influence ornamentation. For instance, when food is abundant and competition is low, one parent may be sufficient to raise the offspring, allowing the other to desert and find a new partner to breed with, thus promoting polygamy and an intensification of sexual selection pressures (Székely and Cuthill 1999). However, we have no evidence that rainfall was correlated with the breeding system, which suggests that the effect we found of the interaction between sex and breeding system on ornamentation of Kentish plovers is not confounded by a potential collinearity with rainfall. Other environmental factors, however, may also play a role in determining the breeding system. In Kentish plovers, high predation rates, for example, may constrain brood desertion, leading to more frequent biparental care and lower levels of polygamy (Amat et al. 1999). The length of the season during which successful breeding may take place is also known to influence the breeding system. A shorter breeding season due to a brief peak in food availability, for example, means that there are fewer opportunities to produce multiple clutches with the same partner than in a population where breeding is potentially spread out over several months. A short breeding season may thus be expected to promote a polygamous mating system, whereas prolonged breeding may be associated with monogamy (García-Peña et al. 2009).

Finally, our dataset included both island and mainland populations, with monogamous populations being restricted to islands, whereas polygamous populations were found on the mainland. Island and mainland populations may exhibit differences in a number of fundamental biological processes, including dispersal strategies, breeding densities, prevalence of parasites and genetic differentiation (Petrie and Kempenaers 1998; Covas 2012; Küpper et al. 2012). Island populations are, for example, expected to exhibit limited dispersal, which is associated with longer-term pairbonds and thus increased levels of monogamy. Consequently, and consistent with our results, island populations are predicted to exhibit less sexual dimorphism (Badyaev and Hill 2003; Roulin and Salamin 2010; but see Doucet et al. 2004). In this study, we were unable to separate the effects of biogeography and breeding system. Future research is therefore needed to establish to what extent biogeographic settings, such as insularity, influence breeding systems and ornamentation.
